# Conservative treatment for high-risk NMIBC failing BCG treatment: who benefits from adding electromotive drug administration (EMDA) of mitomycin C (MMC) to a second BCG induction cycle?

**DOI:** 10.1007/s00345-023-04372-5

**Published:** 2023-03-27

**Authors:** Gian Maria Busetto, Marco Finati, Marco Chirico, Francesco Cinelli, Nicola D’Altilia, Ugo G. Falagario, Francesca Sanguedolce, Francesco Del Giudice, Ettore De Berardinis, Matteo Ferro, Felice Crocetto, Angelo Porreca, Luca Di Gianfrancesco, Beppe Calo’, Vito Mancini, Carlo Bettocchi, Giuseppe Carrieri, Luigi Cormio

**Affiliations:** 1 Department of Urology, University of Foggia, Policlinico Riuniti, Via Luigi Pinto, 1, 71122 Foggia, Italy; 2 Department of Pathology, University of Foggia, Policlinico Riuniti, Foggia, Italy; 3 Department of Urology, Sapienza Rome University, Policlinico Umberto I, Rome, Italy; 4 Department of Urology, European Institute of Oncology (IEO)-IRCCS, Milan, Italy; 5 Department of Urology, University of Naples Federico II, Naples, Italy; 6 Oncological Urology, Veneto Institute of Oncology (IOV)-IRCCS, Padua, Italy; 7 Bonomo Teaching Hospital, Andria, BAT Italy

**Keywords:** Bladder cancer, EMDA, Instillation, Adjuvant therapy, High-risk

## Abstract

**Purpose:**

Radical cystectomy (RC) is the standard treatment for high-risk non muscle-invasive bladder cancer (NMIBC) failing first BCG treatment. A second BCG course is an option for those patients who refuse RC or are not eligible for it, but its success rate is quite low. Aim of the present study was to determine whether the addition of intravesical electromotive drug administration of mytomicin-C (EMDA-MMC) improved the efficacy of second BCG course.

**Methods:**

Patients with high-risk NMIBC having failed first BCG treatment and having refused RC were offered a second BCG induction course either alone (group A) or combined with EMDA-MMC (group B). Recurrence-free survival (RFS), progression-free survival (PFS) and cancer-specific survival (CSS) were tested.

**Results:**

Of the 80 evaluable patients, 44 were in group A and 36 in group B; median follow-up was 38 months. RFS was significantly worse in group A whereas there was no difference in PFS and CSS between the two groups. Stratifying by disease stage, Ta patients receiving combined treatment had statistically better RFS and PFS survival than those receiving BCG only; this difference did not apply to T1 patients. Multivariable analysis confirmed that combined treatment was a significant predictor of recurrence and was close to predict progression. No tested variable was predictive of recurrence or progression in T1 tumours. Among those who underwent RC, CSS was 61.5% in those who had progression and 100% in those who remained with NMIBC.

**Conclusion:**

Combined treatment improved RFS and PFS only in patients with Ta disease.

**Supplementary Information:**

The online version contains supplementary material available at 10.1007/s00345-023-04372-5.

## Introduction

Bladder cancer (BC) is one of the most challenging urological malignancies to manage. Non-muscle-invasive bladder cancer (NMIBC) accounts for almost 75% of all BCs and stratification into risk groups is essential in planning treatment and predicting the risk of recurrence and progression [1]. High- and very high-risk NMIBC include all T1 tumours, Ta high grade (HG) tumours, all concomitant/solitary Carcinoma in situ (Cis), Ta low/high grade with 2/3 additional risk factors [1]; according to the European Association of Urology (EAU) risk tables, the 5-year progression for these patients ranges from 9.6 to 44% [1].

Intravesical Bacillus Calmette-Guérin (BCG) is the standard adjuvant treatment for high-risk NMIBC after trans-urethral resection of bladder tumour (TURBT) [1–3]. The best treatment schedule should include induction followed by maintenance, with no statistically significant difference in recurrence and progression for the 1-year or 3-year maintenance schedule [4, 5].

BCG failure is defined as any HG and/or high-risk disease occurring during or after BCG therapy. Different categories of BCG failures have been identified and are important to better understand outcomes of further conservative treatments. BCG-relapsing tumours are those recurring only after completion of maintenance, BCG-unresponsive include those recurring within 6 months from the beginning of adjuvant BCG, while BCG-refractory are those recurring within 3 months [6, 7]. The gold standard treatment option for BCG failure is radical cystectomy (RC), whereas the other available conservative treatments are considered oncologically inferior [1]. A second BCG course is the standard conservative treatment but in the last few years, alternative treatments such as device-assisted chemotherapy instillations and the electromotive drug administration (EMDA) have been proposed. Combined intravesical chemotherapy with EMDA-mytomicin C (EMDA-MMC) and intravesical immunotherapy with BCG has been suggested to increase anticancer effect [8]. A randomized controlled trial including 212 patients with primary high-risk NMIBC demonstrated that sequential EMDA-MMC and BCG intravesical administration significantly improved the disease-free interval as compared to BCG alone. The authors suggested bladder permeability to be affected by the BCG-induced inflammation up to favouring the electrostimulated mitomycin to better reach the target and exert its anticancer effect [8]. Another research group recently analysed the role of EMDA-MMC alone in high-risk NMIBC failing first BCG treatment and pointed out that such treatment may represent a valid second-line conservative tool in selected patients [9, 10].

The present study therefore aimed to determine whether the addition of intravesical electromotive drug administration of mytomicin-C (EMDA-MMC) improved the efficacy of a second BCG cycle in high-risk NMIBC who failed first BCG treatment.

## Materials and methods

Our prospectively maintained database of NMIBC was queried to identify patients with high-risk NMIBC who failed first BCG treatment, refused RC and therefore underwent a second BCG cycle with or without intravesical EMDA-MMC from January 2014 to February 2021.

According to our policy, all patients having completed the first BCG induction cycle underwent bladder biopsies [11] to assess treatment efficacy. In the absence of recurrent HG disease or CIS, patients were scheduled for BCG maintenance with a monthly BCG instillation for at least 1 year. Those with recurrent HG disease at bladder biopsies or during follow-up were considered BCG failures and primarily offered RC. Those who refused or were not eligible for such treatment were assigned to receive conservative intravesical treatment with BCG alone (group A) or sequential EMDA-MMC and BCG (group B) on the basis of the referring consultant. The BCG only re-induction cycle was carried out as the first BCG course; sequential treatment consisted of a 9-weekly course with two BCG doses followed by a third one with EMDA-MMC (41 mg MMC in 100 ml normal saline, Physion EMDA^®^) instillation. The mytomicin solution was administered through a 16F electrode-catheter inserted into the bladder, after removing residual urine. Two dispersive electrodes were placed on the sides of the navel and a microcurrent applied for 20 min. At the end of the procedure, the bladder was emptied, and the catheter removed.

Like for the first induction cycle, systematic bladder biopsies were performed after the re-induction cycle; if negative or yielding low-grade Ta disease, patients underwent maintenance, which consisted in monthly instillations of BCG for the BCG-only group, or the sequence of EMDA-MMC for the first two months followed by BCG for the third month, for sequential treatment. In both cases, maintenance was carried out for at least 1 year.

Follow-up included cystoscopy and urine cytology every three months, while chest-abdomen computed tomography (CT) was performed once a year. Patients who did not completed the entire re-induction course and those not willing to participate in the study were excluded.

All surgical specimens were processed, described, and reviewed by two dedicated uro-pathologists. For urothelial cancer, grade was classified according to the WHO/ISUP 2016 grading system [2]. Pathological stage was re-assigned following the current American Joint Committee on Cancer 2017 TNM staging system (VIII edition) [3].

### Statistical analysis

The primary endpoint was to assess recurrence-free survival (RFS) and progression-free survival (PFS) of the two treatment options. Recurrence was defined as NMIBC at TURBT during follow-up, whereas progression was defined ≥ T2 BC at TURBT or distant metastases.

For descriptive statistics, continuous variables were reported using median and interquartile range (IQR) whereas categorical variables were reported as percentage; the ANOVA and the chi-square tests were used to examine differences in continuous and categorical variables, respectively.

RFS and PFS of the two treatment options was analysed by Kaplan–Meier estimates after stratification for disease stage. The impact of different variables on RFS and PFS was then assessed by multivariable Cox regression analysis.

All tests were two-sided and statistical significance was defined as *p* value < 0.05. All analyses were performed using SPSS Statistics© 25 (SPSS, IBM Corporation, Armonk, NY, USA).

### Ethics issue

All procedures performed in studies involving human participants were in accordance with the ethical standards of the institutional and/or national research committee and with the 1964 Helsinki Declaration and its later amendments or comparable ethical standards. This study was approved by IRB of Policlinico Riuniti of Foggia on 15th of March 2022 (Approval Code 31/CE/2022). All patients signed an informed consent for the procedure.

## Results

Overall, 83 patients entered the study. Two patients (one for each group) discontinued treatment due to recurrent urinary tract infections, while a third one exhibited intolerance to electromotive administration and continued intravesical treatment with BCG only. All three of them were excluded from the study. Mild skin burns occasionally occurred at first course with EMDA on the electrode’s application side and were treated with moisturizing cream. None of these patients had to postpone or stop the treatment. Therefore, the final cohort consisted of 80 patients, 44 (55%) in group A and 36 (45%) in group B. Their characteristics are summarised in Supplementary Table 1. Patients’ median age was 72 years (IQR 62–79); sixty-eight (85%) were males and 12 (15%) females. Thirty (38%) had a Ta while 50 had a T1 disease; concomitant carcinoma in situ (CIS) was present in 12 (15%) patients, 3 with Ta and 9 with T1 disease. HG Ta occurred in 55.6% patients of group A versus 22.7% in group B (*p* value: 0.003). Twenty-three (29%) patients met the EAU criteria [1] for BCG unresponsive disease whereas the other 57 (71%) were BCG refractory tumours.

At a median follow-up of 38 months (20–58), recurrent low-grade NMIBC occurred in 14/80 (17.5%) patients, recurrent HG NMIBC in 67.5% (54/80) and progression to MIBC in 18.8% (15/80). Therefore, the disease-free rate was and 12/80 (15%); median disease-free time was 12 months (8–24). Overall, 21 (27.3%) patients underwent RC, due to progression to MIBC in 13, or recurrent high-risk/very high-risk NMIBC in 8. Pathologic upstage to extravesical disease (T stage > 3) and presence of nodal metastases occurred in 6/21 (29%) and 7/21 (33%) patients, respectively, and all were found in those having undergone RC for progression to MIBC.

Of the remaining 33 patients with recurrent HG disease, 26 underwent a new induction cycle with BCG, one underwent systemic chemotherapy after detection of lung metastases, and 6 refused any further treatment.

**Fig. 1:**
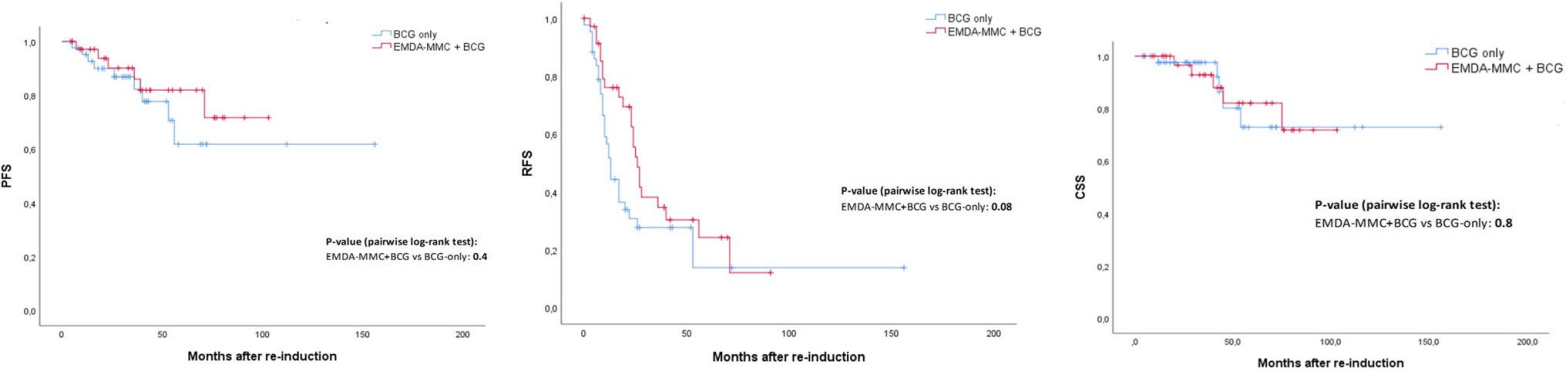
Kaplan–Meier curves of RFS (**A**), PFS (**B**) and CSS (**C**) according to intravesical treatment

Eleven patients (14.3%) died during follow-up with 9 of them dying for BCa; therefore, overall survival (OS) was 85.7% while cancer-specific survival (CSS) was 88.3%. Of the 9 patients who died for BC, 6 belonged to group A and 3 to group B, but this difference in CSS was not statistically significant (Fig. 1); five belonged to those having undergone RC for MIBC whereas none of patients having undergone RC for recurrent HG NMIBC died of BC.

Recurrence rate and progression rate were higher in group A than in group B (71% 31/44 vs. 64% 23/36, *p = *0.7; 21% vs. 17%, *p = *0.5; respectively). Also low-grade recurrence rate was higher in group A than in group B (20% vs. 14%, *p = *0.4). Kaplan–Meier estimates for RFS (Fig. 1) showed a clear advantage for sequential treatment, which was close but did not reach statistical significance (*p = *0.08) for recurrent HG disease whereas it was statistically significant when also low-grade recurrences were considered (*p = *0.03). Conversely, difference in PFS (Fig. 1) was not statistically significant.

**Fig. 2:**
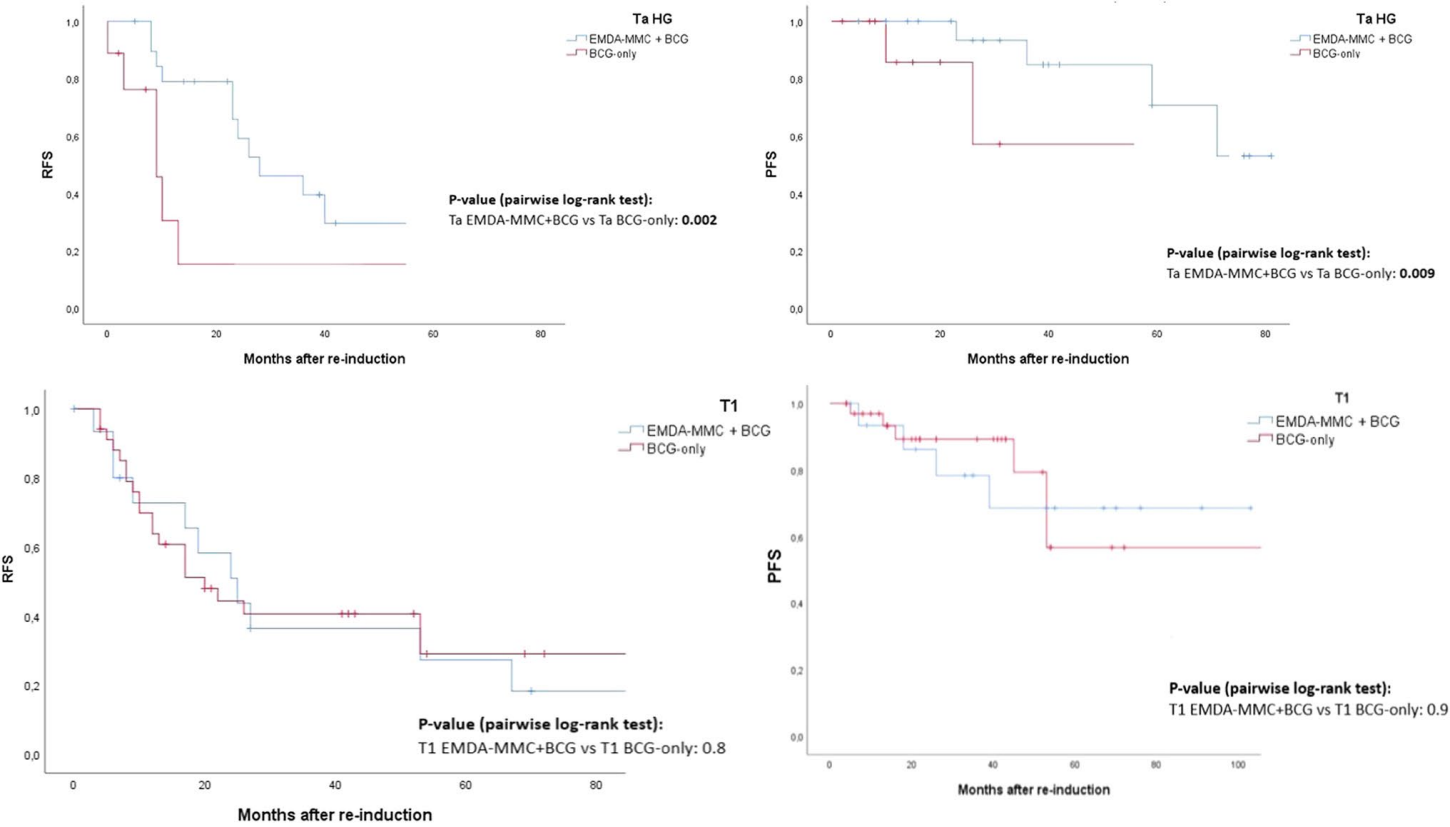
Kaplan–Meier curves of RFS (**A**), PFS (**B**) in: **a** Ta patients according to intravesical treatment, **b** T1 patients according to intravesical treatment

When stratified for disease stage, namely Ta vs. T1 disease, the curves pointed out that Ta patients (Fig. 2) receiving sequential treatment had statistically better RFS and PFS survival than those receiving BCG only (all *p* values < 0.009); this difference did not apply to T1 patients (Fig. 2).

Multivariable analysis (Table 1) confirmed that, in Ta patients, treatment was a significant predictor of recurrence, together with age, female gender, tumour multifocality and presence of concomitant CIS. No tested variable predicted recurrence in T1 tumours, and none predicted progression in both Ta and T1 tumours, although treatment was close but did not reach statistical significance (*p = *0.06) in this respect, probably due to the low number of events.

**Table 1:** Multivariable Cox regression analysis predicting recurrence and progression according to pathologic stage

Recurrence	Ta		T1	
	HR (CI 95%)	*p* value	HR (CI 95%)	*p* value
Age (years)	0.9 (0.80–0.95)	**0.001**	1.0 (0.94–1.02)	0.4
Gender (female vs male)	4.1 (3.19–5.28)	**0.004**	3.5 (0.77–15.81)	0.1
Primary versus recurrent BC	4.3 (0.81–23.02)	0.09	2.5 (0.82–7.80)	0.1
Single vs multifocal	0.01 (0.006–0.61)	**0.02**	1.6 (0.60–4.11)	0.4
Maximum tumour size				
> 3 cm vs < 3 cm				0.6
Concomitant CIS: yes vs no	2.4 (0.50–11.54)	0.3	1.3 (0.51–3.18)	0.9
Type of re-induction	4.5 (2.57–8.02)	**0.009**	0.9 (0.28–2.99)	
BCG + EMDA-MMC vs BCG only	0.01 (0.001–0.09)	** < 0.001**	1.9 (0.73–5.05)	0.2

Progression	Ta		T1	
	HR (CI 95%)	*p* value	HR (CI 95%)	*p* value
Age (years)	0.9 (0.76–1.05)	0.2	1.0 (0.96–1.08)	0.6
Gender (female vs male)	0.1 (0.01–2.46)	0.2	0.5 (0.04–8.29)	0.7
Primary versus recurrent BC	1.4 (0.21–5.24)	0.2	4.4 (0.48–41.22)	0.2
Single vs multifocal	3.3 (0.17–6.16)	0.2	2.9 (0.51–16.30)	0.2
Maximum tumour size				
> 3 cm vs < 3 cm	0.7 (0.01–9.99)	0.8	1.5 (0.30–7.52)	0.6
Concomitant CIS: yes vs no	2.0 (0.06–67.04)	0.7	2.7 (0.42–17.81)	0.3
Type of re-induction				
BCG + EMDA-MMC vs BCG only	0.01 (0.001–1.45)	0.06	1.9 (0.36–10.61)	0.4

In bold statistically significant values

## Discussion

Patients with high-risk NMIBC failing first BCG treatment are unlikely to respond to a second BCG course unless they are late-relapsing cases [9, 12]. In the past, attempts to find alternative treatments to a second BCG course have been oriented towards the use of cytotoxic intravesical therapies [13–15] such as gemcitabine and valrubicin, but with little success.

Promising data were recently reported using novel immunotherapies. Systemic pembrolizumab achieved a 40% complete response rate in a prospective phase II study which was maintained in 48% of patients for up to 12 months (*n = *101), resulting in FDA approval of the study drug for this patient population [16]. Similarly, a phase III multicentre RCT using intravesical nadofaragene firadenovec demonstrated complete response in 53.4% of patients with BCG-unresponsive CIS [17].

Even greater attention has been paid to novel device-assisted intravesical treatments. The possibility to treat BCG failures with intravesical EMDA-MMC has recently been explored by Racioppi et al. in 26 patients. At the end of 3 years follow-up, 6 patients (61.5%) preserved their native bladder; 10 patients (38.4%) underwent radical cystectomy, in 6 patients (23.1%) for recurrent HG NMIBC and in 4 patients (15.4%) for progression to muscle-invasive disease. Disease-free survival was significantly longer for Ta tumours as compared to T1 tumours and those with concomitant CIS (*p* value < 0.05) [9]. In a subsequent study, Di Gianfrancesco et al. analysed 209 consecutive patients with HG NMIBC having failed BCG treatment; 107 refused RC and were offered electromotive drug administration (*n = *44) or chemohyperthermia (*n = *63) (group A), whereas 102 underwent RC (group B). At median follow-up of 59 months, group A and B showed similar overall survival rates (91.6% vs. 90.2%; *p > *0.05.) and cancer-specific survival rates (94.4% vs. 96.1%; *p > *0.05) but significant differences in HG disease-free survival rates (43% vs. 74.5%; *p < *0.05) and progression-free survival rates (59.8% vs. 75.5%; *p < *0.05) [10].

In a recent phase III RCT 104 patients with high-risk NMIBC having failed first BCG treatment were randomised to receive radiofrequency-induced thermo-chemotherapy (RITE) or a second BCG course. There was no significant difference in DFS between treatment arms in non-CIS patients whereas in CIS patients DFS was significantly lower in those who received RITE as compared to those receiving a second BCG course. Adverse events and health-related quality of life between treatment arms were comparable [18].

The possibility to treat high-risk NMIBC failing first BCG treatment by sequential EMDA-MMC + BCG was recently addressed by Juvet et al. [19]. In the 26 patients they treated, PFS rates were 58.3% at 1 year and 48.9% at 2 years; RFS was 41.9% at 1 year and 27.2% at 2 years. Side effects included dysuria (19.2%), haematuria (19.2%), and frequency (11.5%). Three patients were admitted for side effects but managed conservatively.

To our knowledge, the present study is the first comparing the efficacy of second BCG treatment with that of a sequential intravesical instillations of EMDA-MMC and BCG in the setting of HG NMIBC failing BCG treatment. The Kaplan–Meier curves depicting RFS and PFS following the two treatment options pointed out that sequential treatment improved RFS but had no effect on PFS. When stratified for disease stage, namely Ta vs. T1 disease, the curves pointed out that Ta patients receiving sequential treatment had statistically better RFS and PFS survival than those receiving BCG only; this difference did not apply to T1 patients. Multivariable analysis confirmed that type of treatment was a significant predictor of recurrence in Ta but not in T1 tumours. Moreover, it was the only factor that was close to predict progression of Ta tumours (*p = *0.06), though it did not reach statistical significance probably due to the low number of events. Indeed, less than 20% of patients progressed to muscle-invasive disease over the study time, two/thirds kept their bladder and CSS was as high as 88%. Interestingly, and differently from previous series, concomitant CIS did not negatively impact on progression. As for safety, most of side effects were linked to the use of BCG rather than to EMDA.

Taken together, our findings suggest that sequential treatment reduced disease recurrence and progression with such benefits being more evident in Ta disease. Reducing the risk of progression remains a critical issue; indeed, 38.5% of patients having undergone RC for progression died for BCa despite radical treatment whereas none of patients having undergone RC for recurrent NMIBC died for BCa. Our findings reinforce the concept that RC should be the standard treatment for BCG failures; having said this, RC has a well-known potential morbidity and mortality that should be balanced with the potential benefits of bladder salvage. Moreover, the finding that nearly 40% of patients having undergone RC for progression anyway died for BCa strongly points at the role of tumour biological features and reinforces the concept that the identification of biomarkers predicting response to available conservative treatments remains a critical issue [20–23].

Although promising results emerged, the retrospective nature of our study limits the strengths of our study. Non-randomised matching resulted in a greater percentage of HG Ta tumour in sequential arm as potential bias in assessing a definitive advantage of this treatment over BCG-only. Another potential study limitation is not having tested the role of biomarkers in predicting disease outcome after such treatments. Another pitfall could be having offered such treatments only to patients having refused RC rather than to all BCG failures.

## Conclusions

Treating high-risk NMIBC patients failing first BCG treatment is a major clinical issue. The present study pointed out that the addition of EMDA-MMC to second BCG treatment reduced disease recurrence and progression but such benefits were relevant only in patients with Ta disease. Te overall progression rate was less than 20% but CSS was 61.5% in patients who had progressed as opposed to 100% in those who had not. These findings could be relevant in counselling patients with this challenging clinical condition.

## Supplementary Information

Below is the link to the electronic supplementary material.

Supplementary file1 (DOCX 15 KB)

## Data Availability

The data that support the findings of this study are available from the corresponding author, [G.M.B.], upon reasonable request.
