# Supplementary material for: Conservative treatment for high-risk NMIBC failing BCG treatment: who benefits from adding electromotive drug administration (EMDA) of mitomycin C (MMC) to a second BCG induction cycle?
Source: World J Urol. 2023 Mar 27;41(5):1329–35. doi: 10.1007/s00345-023-04372-5 (PMC10188396; doi:10.1007/s00345-023-04372-5)
Supplement: Supplementary file 1 — Supplementary file1 (DOCX 15 KB) [file 345_2023_4372_MOESM1_ESM.docx]

**Supplementary Table 1.** Patients’ characteristics according to treatment.

| N= 80 | BCG + EMDA-MMC (n= 36; 45%) | BCG-only  (n=44; 55%) | P-value |
| --- | --- | --- | --- |
| Age (Years,IQR) | 73 (65-80) | 71 (62-76) | 0.5 |
| Gender (%):  - Male  - Female | 28 (77.8)  8 (22.2) | 40 (90.9)  4 (9.1) | 0.1 |
| Smoking Habit:  - None  - Former smoker  - Current smoker | 10 (27.8)  8 (22.2)  18 (50.0) | 12 (27.3)  12 (27.3)  20 (45.5) | 0.8 |
| Previous history of LG BC:  - Primary  - Recurrent | 12 (33.3)  24 (66.7) | 10 (22.7)  34 (77.3) | 0.3 |
| Pathologic Stage (%)  - Ta  - T1 | 20 (55.6)  16 (44.4) | 10 (22.7)  34 (77.3) | **0.003** |
| Concomitant CIS:  Yes  No | 32 (88.9)  4 (11.1) | 36 (81.8)  8 (18.2) | 0.4 |
| Maximum tumour size:  < 3 cm  > 3 cm | 28 (77.8)  8 (22.2) | 28 (63.6)  16 (36.4) | 0.2 |
| Number of tumour:  - Single  - Multifocal | 20 (55.6)  16 (44.4) | 15 (34.1)  29 (65.9) | 0.06 |
| Median follow-up (months, IQR) | 41 (24-69) | 33 (19-54) | 0.5 |
